# Resistance to *Bacillus thuringiensis* toxin Cry2Ab and survival on single‐toxin and pyramided cotton in cotton bollworm from China

**DOI:** 10.1111/eva.12438

**Published:** 2016-12-16

**Authors:** Laipan Liu, Meijing Gao, Song Yang, Shaoyan Liu, Yidong Wu, Yves Carrière, Yihua Yang

**Affiliations:** ^1^ College of Plant ProtectionNanjing Agricultural UniversityNanjingChina; ^2^ Department of EntomologyUniversity of ArizonaTucsonAZUSA

**Keywords:** Bt cotton, cross‐resistance, dominance of resistance, fitness costs, pyramided crops, redundant killing, resistance management

## Abstract

Evolution of *Helicoverpa armigera* resistance to *Bacillus thuringiensis* (Bt) cotton producing Cry1Ac is progressing in northern China, and replacement of Cry1Ac cotton by pyramided Bt cotton has been considered to counter such resistance. Here, we investigated four of the eight conditions underlying success of the refuge strategy for delaying resistance to Cry1Ac+Cry2Ab cotton, a pyramid that has been used extensively against *H. armigera* outside China. Laboratory bioassays of a Cry2Ab‐selected strain (An2Ab) and a related unselected strain (An) reveal that resistance to Cry2Ab (130‐fold) was nearly dominant, autosomally inherited, and controlled by more than one locus. Strong cross‐resistance occurred between Cry2Ab and Cry2Aa (81‐fold). Weaker cross‐resistance (18‐ to 22‐fold) between Cry2Ab and Cry1A toxins was also present and significantly increased survival of An2Ab relative to An on cotton cultivars producing the fusion protein Cry1Ac/Cry1Ab or Cry1Ac. Survival on Cry1Ac+Cry2Ab cotton was also significantly higher in An2Ab than in An, showing that redundant killing on this pyramid was incomplete. Survival on non‐Bt cotton did not differ significantly between An2Ab and An, indicating an absence of fitness costs affecting this trait. These results indicate that a switch to three‐toxin pyramided cotton could be valuable for increasing durability of Bt cotton in China.

## 1 Introduction

*Bacillus thuringiensis* (Bt) cotton producing the toxin Cry1Ac has provided significant environmental and economic benefits since its introduction in China in 1997 for control of major lepidopteran pests (Huang, Rozelle, Pray, & Wang, 2002; Lu, Wu, Jiang, Guo, & Desneux, 2012; Wu & Guo, 2005; Wu, Lu, Feng, Jiang, & Zhao, 2008). Nevertheless, the frequency of resistance to Cry1Ac has regularly and significantly increased in populations of two target pests, *Helicoverpa armigera* and *Pectinophora gossypiella,* indicating that replacement of Cry1Ac cotton with pyramided Bt cotton is needed to counter the threat of resistance (Jin et al., 2015; Tabashnik, Wu, & Wu, 2012; Wan et al., 2012; Wu, 2014; Zhang et al., 2011). Because Cry1Ac+Cry2Ab cotton may provide appropriate control of both pests when the frequency of resistance to at least one of the toxins is low (Downes & Mahon, 2012; Fabrick et al., 2015; Mahon & Olsen, 2009; Tabashnik et al., 2002), this pyramid has been evaluated for replacement of Cry1Ac cotton in China (Gao, Liu, Wu, & Wu, 2015; Tabashnik et al., 2012). Here, we used laboratory experiments to better understand the risk of *H. armigera* resistance to Cry1Ac+Cry2Ab cotton in China, by assessing several of the conditions underlying success of the refuge strategy for delaying resistance to this pyramided Bt crops.

A relatively high number of susceptible *H. armigera* larvae can survive on Cry1Ac+Cry2Ab cotton (Downes et al., 2010; Mahon & Olsen, 2009), showing that this pest has a relatively low inherent susceptibility to these Bt toxins. Relative to pests with high inherent susceptibility to Bt toxins, pests with low susceptibility are less likely to meet conditions underlying success of the refuge strategy (Carrière, Crickmore, & Tabashnik, 2015; Carrière, Crowder, & Tabashnik, 2010; Carrière, Fabrick, & Tabashnik, 2016; Tabashnik, Brévault, & Carrière, 2013; Tabashnik et al., 2009). Specifically, pests with low susceptibility are not expected to exhibit recessive resistance to Bt toxins and redundant killing, which occurs when each toxin in a pyramid kills most or all susceptible insects (Brévault et al., 2013; Carrière et al., 2010, 2015, 2016; Tabashnik, Mota‐Sanchez, Whalon, Hollingworth, & Carrière, 2014; Tabashnik et al., 2013). Furthermore, cross‐resistance between Cry toxins produced in pyramids is pervasive, generally associated with amino acid sequence similarity between toxins in domain II, and most likely to accelerate evolution of resistance in pests with low susceptibility (Carrière et al., 2015, 2016; Welch et al., 2015).

We specifically considered four of the eight conditions (Carrière et al., 2015, 2016) affecting evolution of *H. armigera* resistance to Cry1Ac+Cry2Ab cotton in China: dominance of resistance to Cry2Ab, cross‐resistance between Cry1Ac and Cry2Ab, extent of redundant killing, and fitness costs associated with Cry2Ab resistance. We investigated these conditions by measuring responses of a strain selected for resistance to Cry2Ab in the laboratory (An2Ab), a related unselected strain (An), and relevant crosses between these strains in bioassays involving artificial diets treated with Cry1A and Cry2A toxins and Bt cotton plants producing Cry1Ac, a fusion protein Cry1Ac/Cry1Ab, and Cry1Ac+Cry2Ab. We also investigated the genetic basis of resistance to Cry2Ab (number of loci affecting resistance, maternal effects, and sex linkage) in the An2Ab strain.

## 2 Materials and methods

### 2.1 Insects

The susceptible An strain of *H. armigera* was started in June 2009 from the progeny of more than 100 field‐mated females collected in Anyang, Henan province of northern China. The An strain has been maintained in the laboratory without exposure to Bt toxins or insecticides since collected. Susceptibility of the An strain to Cry2Ab is similar to that of the laboratory GR strain established in the mid‐1980 from field collections in northern New South Wales, Australia (Mahon, Olsen, Garsia, & Young, 2007). The Cry2Ab‐resistant strain originated from a F_2_ screen performed on 104 field‐mated females collected at the same time and location. Each of the 104 females produced an isofemale line, and F_1_ adults from each isofemale line were mated to produce F_2_ progeny. A diet overlay bioassay (described below) was used to test 96 F_2_ neonates from each line at the discriminating concentration of 2 μg Cry2Ab per cm^2^ diet, which was adopted from Mahon et al. (2007). This discriminating concentration killed all larvae tested in the An strain (*n *> 1,000). Survivors from the ten lines with the highest survival (from 6.3% to 16.7%) were pooled to produce the An2Ab strain, which was further selected for resistance to Cry2Ab for 37 consecutive generations. During selection, an average of 1,440 neonates were selected per generation and concentrations of Cry2Ab were chosen to yield 60%–80% mortality. After completion of diet bioassays, survivors were reared to pupation on untreated diet to propagate the An2Ab strain.

Larvae were reared on artificial diet, and adults were maintained as described previously (Zhang et al., 2011). All experiments were conducted at 26°C (±1°C) and 60% (±10%) RH with a photoperiod of 16‐hr L: 8‐hr D.

### 2.2 Bt toxins

The Institute of Plant Protection, Chinese Academy of Agricultural Sciences (CAAS), provided the Cry2Ab protoxin used for selection and in experiments. The Cry1A activated toxins (Cry1Aa, Cry1Ab, and Cry1Ac) and Cry2Aa protoxin used in experiments were provided by Dr. Marianne P. Carey (Case Western Reserve University, USA). Cry2Ab was produced as outlined in Wei et al. (2015), and Cry1Aa, Cry1Ab, Cry1Ac, and Cry2Aa were produced according to Monnerat et al. (1999).

### 2.3 Bioassays

We used diet overlay bioassays for selection of resistance to Cry2Ab and evaluation of *H. armigera* responses to the Bt toxins. Cry1A toxins were solubilized in 100 mM Na_2_CO_3_ buffer (pH 10.3, containing 10 mM DTT) and Cry2A protoxins in 50 mM Na_2_CO_3_ buffer (pH 12.1, containing 5 mM EDTA and 10 mM EGTA) to produce toxin stock suspensions (1 mg/ml) for each Bt toxins. Stock suspensions were further diluted with a 10 mM, pH 7.4 phosphate‐buffered solution (PBS) to obtain appropriate concentrations used in bioassays. Liquid artificial diet (900 μl) was dispensed into each well of a 24‐well plate. After the diet cooled and solidified, 100 μl of the PBS solution with the desired concentration of Bt toxin was applied evenly to the diet surface of each well of a 24‐well plate and allowed to air dry at room temperature. A single larva was placed in each well of the plate and covered with two layers of black cloth to prevent escape. Forty‐eight larvae (two replicates) were tested for each strain and toxin concentration, including a control with PBS and no toxin. At the end of the bioassay period, response of larvae was scored as dead if they died or weighed <5 mg.

For bioassays with the Cry2Aa and Cry2Ab protoxins, unfed neonates (24 hr old) were used and the response of larvae was recorded after 7 days. This method was established in Australia for testing Cry2Ab against *H. armigera* (Mahon et al., 2007; Welch et al., 2015). For bioassays with Cry1A toxins, second instars starved for 4 hr were tested and the response of larvae was recorded after 5 days, as in our previous studies (Jin et al., 2013; Xu, Yu, & Wu, 2005; Yang, Chen, Wu, Yang, & Wu, 2007; Zhang et al., 2011).

### 2.4 Inheritance of resistance to Cry2Ab

Male moths from the An2Ab strain were mass‐crossed with virgin females of the An strain and vice versa. F_1_ hybrids were backcrossed to the An strain because resistance to Cry2Ab was incompletely dominant. At least 50 adults of each sex were used in mass crosses. Responses to Cry2Ab of An2Ab, An, and F_1_ hybrids from the two reciprocal crosses and the backcross progeny were determined using the bioassay described earlier.

We calculated the dominance parameter *h*, which varies from 0 (completely recessive) to 1 (completely dominant) (Liu & Tabashnik, 1997), using survival (%) at the diagnostic concentration of 2 μg Cry2Ab per cm^2^ of diet as follows: *h *= (survival of F_1 _− survival of An)/(survival of An2Ab − survival of An). We used the EC_50_ values (concentration of toxin causing 50% of larval response) of An2Ab, An, and F_1_ progeny to calculate the dominance parameter *D* (Stone, 1968), which ranges from −1 (completely recessive) to 1 (completely dominant).

The number of loci conferring resistance was assessed using three methods: (1) direct test of one‐locus model (Georghiou, 1969; Tabashnik, 1991), (2) estimation of number of loci affecting resistance using models with one, two, and five loci (Tabashnik, 1991), and (3) minimum number of loci involved in resistance (Lande, 1981). For the first two methods, we assumed that each locus has one allele for susceptibility and one allele for resistance. In the first method, the expected mortality in the F_1_ × An backcrossed at concentration c is M_c_ **= 0.5 × (M_F1_ + M_An_), where M_F1_ and M_An_ are mortalities of the F_1_ offspring and An strain, respectively. In the second method, we assumed that effects of alleles at all loci are equal and additive on a logarithmic scale and independent segregation of resistance loci to calculate expected responses of the backcross progeny (Tabashnik, 1991). For the Lande (1981) method, the minimum number of loci involved in resistance (*n*_e_) is *n*_e_ **= (μ_P2_ − μ_P1_)^2^/(8σ_s_^2^) ≤ *n*, where μ_P2_ and μ_P1_ are the log (EC_50_) for resistant and susceptible strains, respectively. The extra genetic variance segregating in the backcross generation beyond that of F_1_ hybrids, σ_s_^2^, was estimated using equation (2) in Tabashnik, Schwartz, Finson, and Johnson (1992) and data from the dose–response curves of the backcross (F_1_ × An), F_1_, An, and An2Ab strains (Tabashnik, 1991).

### 2.5 Survival on Bt cotton and non‐Bt cotton plant materials

Four cotton cultivars were planted in the field on April 17, 2014, at Luhe in the Jiangsu province of China. Cultivars were GK19 producing the fusion protein Cry1Ac/Cry1Ab, its non‐Bt cotton parent cultivar Simian3 (Wan, Zhang, Wu, & Huang, 2005), 33B producing Cry1Ac toxin (Bollgard), and a dual Bt cotton variety (Bollgard 2) producing Cry1Ac toxin and Cry2Ab protoxin (Knight, Head, & Rogers, 2013). No insecticides were used to protect cotton plants. Insect feeding experiments were started on August 6, 2014, when cotton plants were bearing bolls.

Feeding experiments were carried out in growth chambers using cotton material from field‐grown plants. Neonates (up to 24 hr old) of the An and An2Ab strains were initially fed cotton leaves (top second or third leaf on the main stem) for 5 days. For each combination of strain and cultivar, groups of five neonates (*n *= 30 groups; total 150 neonates per combination) were put on a cotton leaf kept in a 115‐ml glass tube. To preserve leaf turgor, the petiole of each leaf was inserted into 20 ml of 1% agar gel at the bottom of the tube. After transfer of neonates, each tube was covered with two layers of black cloth to prevent insects from escaping.

After the initial 5‐day period, survivors (either second or third instars) from tubes were transferred to a 330‐ml clear plastic cups covered with two layers of black cloth. Larvae were supplied with cotton stems bearing leaves, buds, and bolls until they reached the fourth instar. Plant stems were inserted into 40 ml of 1% agar gel at the bottom of cups. For each combination of strain and Bt cultivar, 30 cups with plant material were arbitrarily divided into three groups (*n *= 10 cups per group), and each group was located at an arbitrarily selected location on a shelf in a growth chamber. Because survival after 5 days on non‐Bt cotton was high (near 100%), for each strain, survivors from each of six arbitrarily sampled tubes (i.e., 20% of tubes) were transferred to cups with non‐Bt cotton plant material (*n *= 6 cups per strain). The six cups were arbitrarily divided into three groups (*n *= 2 cups per group), and each group placed at an arbitrarily selected location on a shelf in a growth chamber.

Once the larvae in cups reached fourth instar, they were transferred individually to cups containing a stem bearing leaves, buds, and bolls to prevent cannibalism. Plant material was replaced every 7 days, and survival was recorded once a week until pupation.

### 2.6 Toxin concentration in plants

On July 28 and August 30 of 2014, Bt cotton leaves, buds, and bolls were collected from plants and stored at −80°C for subsequent determination of Cry1Ac and Cry2Ab concentrations. On each date, one sample for each plant structure was collected from each of 40 plants. For each plant structure and cultivar, three or four samples (*n *= 10 per sample) were arbitrarily taken across dates. These samples were analyzed for concentration of Cry1A and Cry2Ab toxins using toxin‐specific enzyme‐linked immunosorbent assays (ELISA). As in Greenplate et al. (2003), the concentration of Cry1Ac or Cry1Ab/Cry1Ac was measured with a QualiPlate™ Kit for Cry1Ab/Cry1Ac (Envirologix, Portland, ME, USA) and the concentration of Cry2Ab was measured with a QuantiPlate™ Kit for Cry2A (Envirologix, Portland, ME, USA).

### 2.7 Data analysis

For each strain tested against each Bt toxin, we used probit analysis (LeOra Software 2002) to estimate the concentration causing 50% of larval response (EC_50_), the 95% fiducial limits of the EC_50_, the slope of the concentration‐response line, and the standard error of the slope. We considered two EC_50_ values significantly different if their 95% fiducial limits did not overlap, which is a conservative criterion (Payton, Greenstone, & Schenker, 2003).

To assess the fit of models evaluated with the first two methods, we used a goodness‐of‐fit test that considered overall deviation between observed and expected mortality across Cry2Ab concentrations (Tabashnik, 1991). For the second method, we also calculated the absolute difference between observed and expected mortality (%) for each concentration and model. Multiple regression followed by linear contrasts (SAS Institute 2013) was used to compare the average absolute difference between observed and expected mortality (log X + 1‐transformed) among models, after correcting for effects of concentration (Crowder et al., 2009).

Logistic regression for binomial counts (SAS Institute 2013) was used to evaluate effects of strain, cultivar, and the interaction between these factors on the odds of *H. armigera* survival from neonate to pupation. Linear contrasts were used to compare survival of the An and An2Ab strain on each cultivar.

Two‐way ANOVA (SAS Institute 2013) was used to assess effects of Bt cultivars, plant structures (i.e., buds, bolls, and leaves), and the interaction between these factors on the concentration of Cry1A toxins. Linear contrasts were used to compare Cry1A concentrations between Bt cultivars. For Cry1Ac+Cry2Ab cotton, we used one‐way ANOVA to compare Cry2Ab concentration among plant structures.

## 3 Results

### 3.1 Inheritance of resistance to Cry2Ab

**Figure 1:**
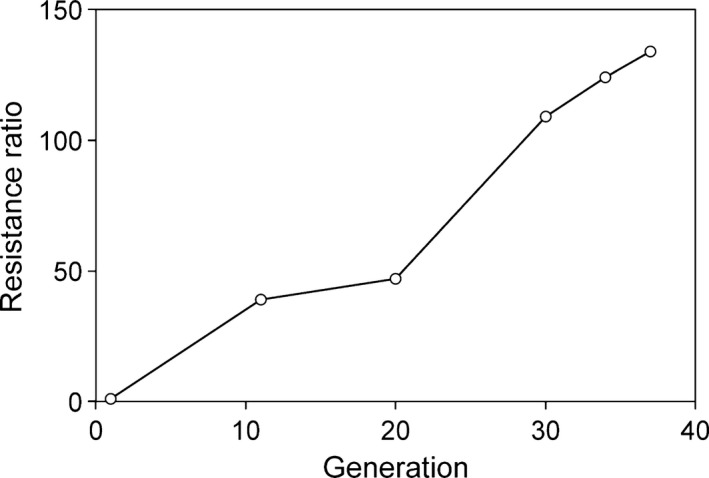
Development of resistance to Cry2Ab of the An2Ab strain of *Helicoverpa armigera* under selection with Cry2Ab. The resistance ratio was calculated as EC_50_ An2Ab/EC_50_ An

Relative to the EC_50_ of the susceptible An strain, Cry2Ab resistance in the selected An2Ab strain increased by 39‐fold after 11 generations and reached 130‐fold after 37 generations of selection (Figure 1). The EC_50_ values and slopes of the F_1_ progeny from reciprocal crosses between An2Ab and An were similar (Table 1), indicating an absence of sex linkage and maternal effects affecting resistance. Resistance of the F_1_ progeny (93‐ and 86‐fold) was close to that of the An2Ab strain (130‐fold), with *D* and *h* values near 1 (Table 1). Both parameters show that Cry2Ab resistance in An2Ab was nearly dominant.

**Table 1:** Responses to Cry2Ab for the resistant strain (An2Ab), susceptible strain (An), F_1_ progeny (An2Ab × An), and backcross (F_1_ × An) of *Helicoverpa armigera*

Source	EC_50_ (95% Fiducial limits) (μg/cm^2^)	Slope ± SE	*n*	RR^a^	D^b^	*h* c
Strain						
An	0.07 (0.05–0.08)	1.9 ± 0.2	384	1		
An2Ab	9.1 (7.1–12.4)	1.7 ± 0.2	336	130		
F_1_						
An2Ab♂ × An♀	6.5 (5.0–8.8)	1.6 ± 0.2	384	93	0.86	0.92
An♂ × An2Ab♀	6.0 (4.6–8.4)	1.4 ± 0.2	384	86	0.83	0.90
Backcross						
(An2Ab × An) ♂ × An♀	1.7 (1.2–2.3)	1.1 ± 0.1	528	24		

a RR (resistance ratio)* *= EC_50_ (An2Ab, F_1_ or Backcross) ÷ EC_50_ (An).

b D (dominance of resistance) was calculated using the method of Stone (1968). D values range from −1 (completely recessive) to 1 (completely dominant).

c The dominance parameter *h* varies from 0 (completely recessive) to 1 (completely dominant).

**Figure 2:**
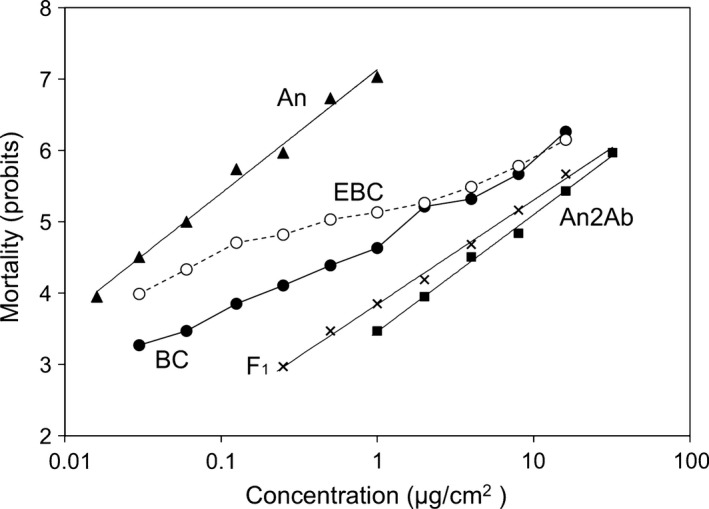
Responses to Cry2Ab of *Helicoverpa armigera* larvae from a susceptible strain (An), a resistant strain (An2Ab), F_1_ progeny (An2Ab ♂ × An♀), and backcross progeny (F_1_♂ × An♀). The backcross curve (BC) shows observed mortality at each concentration. The EBC line shows expected mortality for the backcross progeny calculated with a one‐locus model (method 1)

In direct tests of the one‐locus model (method 1), observed mortality in the backcross progeny (F_1_ × An) differed significantly from expected mortality (Figure 2, *X* ^2^ **= 58.74, *df *= 9, *p *< .0001). With method 2, observed mortality was significantly different from expected mortality for the model with one locus, but there was no significant difference between observed and expected mortality for models with two and five loci (Table S1). The average absolute difference between observed and expected mortality was highest for the one‐locus model (53.1%) and declined for the two‐locus (24.2%) and five‐locus (12.1%) model (Table S1). Both model (*F *= 18.10, *df *= 2, 18, *p *< .0001) and concentration (*F *= 15.22, *df *= 9, 18, *p *< .0001) significantly affected the absolute difference between observed and expected mortality. Linear contrasts revealed that deviations between observed and expected mortality were significantly higher in the one‐locus than two‐locus (*p *= .0012) and five‐locus model (*p *< .0001). Deviations were also significantly higher in the two‐locus than five‐locus model (*p *= .05).

In agreement with these results, the minimum number of independently segregating loci estimated with method 3 was 4.5. This indicates that more than one locus and less than five loci controlled resistance to Cry2Ab in the An2Ab strain.

### 3.2 Cross‐resistance in the An2Ab strain

After 37 generations of selection, cross‐resistance was high between Cry2Ab and Cry2Aa (81‐fold) and lower (18‐ to 22‐fold) between Cry2Ab and the Cry1A toxins (Table 2). The lack of overlap between fiducial limits of An and An2Ab for Cry2Aa, Cry1Ab, and Cry1Ac indicates significant cross‐resistance between Cry2Ab and these toxins. The difference between the EC_50_ of An and An2Ab for Cry1Aa also indicates cross‐resistance between Cry2Ab and Cry1Aa, although low mortality of An2Ab at the highest Cry1Aa concentration tested prevented statistical comparison of these EC_50_s (Table 2).

**Table 2:** Responses to Bt toxins of two strains of *Helicoverpa armigera*: a strain selected in the laboratory with Cry2Ab (An2Ab) and an unselected strain (An)

Strain	Bt toxin	EC_50_ (95% Fiducial limits) (μg/cm^2^)	Slope ± SE	*n*	RR^a^
An2Ab (Selected)	Cry2Ab	9.1 (7.1–12.4)	1.7 ± 0.2	336	130
	Cry2Aa	8.1 (6.4–10.8)	1.8 ± 0.2	336	81
	Cry1Aa	>20^b^	NA	384	>20
	Cry1Ab	10.5 (8.8–13.0)	2.6 ± 0.3	384	18
	Cry1Ac	1.1 (0.9–1.4)	1.6 ± 0.2	384	22
An (Control)	Cry2Ab	0.07 (0.05–0.08)	1.9 ± 0.2	384	
	Cry2Aa	0.1 (0.08–0.1)	1.9 ± 0.2	384	
	Cry1Aa	1.0 (0.8–1.4)	1.3 ± 0.1	384	
	Cry1Ab	0.6 (0.5–0.9)	1.3 ± 0.2	384	
	Cry1Ac	0.05 (0.04–0.07)	1.9 ± 0.2	384	

a RR (resistance ratio)* *= EC_50_ (An2Ab) ÷ EC_50_ (An).

b Mortality was 23% at 20 μg Cry1Aa/cm^2^ diet, the highest concentration tested.

### 3.3 Survival from neonate to pupation on Bt and non‐Bt cotton

The odds of survival to pupation were significantly affected by strain (*X* ^2^ **= 26.43, *p *< .0001), cultivar (*X* ^2^ **= 57.14, *p *< .0001), and the interaction between these factors (*X* ^2^ **= 19.72, *p *= .0002). Survival to pupation was significantly higher in An2Ab than in An on cultivars producing a single Cry1A toxin (GK19 and 33B), showing that cross‐resistance between Cry2Ab and these toxins (Table 2) was sufficient to increase survival of An2Ab on these cultivars (Table 3). Survival to pupation was also significantly higher in An2Ab than in An on Cry1Ac+Cry2Ab cotton (Table 3). This indicates incomplete redundant killing that could arise at least in part from cross‐resistance between Cry2Ab and Cry1Ac (Table 2).

**Table 3:** Survival from neonate to pupation of *Helicoverpa armigera* from an unselected strain (An) and a Cry2Ab‐resistant strain (An2Ab) reared on plant material from non‐Bt cotton and Bt cotton producing a Cry1A toxin or Cry1Ac+Cry2Ab

Cultivar	Strain	Survival % (SE)	*X* ^2^ a	*p* b
Non‐Bt	An	30.0 (5.8)	0.34	.56
	An2Ab	23.3 (3.3)		
33B (Cry1Ac)	An	0	11.31	.00077
	An2Ab	5.3 (1.3)		
GK19 (Cry1Ac/Cry1Ab)	An	0	14.21	.00016
	An2Ab	6.7 (0.7)		
Bollgard 2 (Cry1Ac+Cry2Ab)	An	0	8.44	.0037
	An2Ab	4.0 (2.0)		

a Chi‐square statistics from linear contrasts comparing survival of the strains on each cultivar.

b Probability indicating significant difference (*p *< .05) between survival of the strains on each cultivar.

By contrast, survival on non‐Bt cotton did not differ significantly between An2Ab and An (Table 3), indicating an absence of fitness costs affecting this trait.

### 3.4 Concentration of Cry1A and Cry2Ab toxins in Bt cultivars and plant structures

The concentration of Cry1A toxins differed significantly among Bt cultivars (*F *= 13.69, *df *= 2, 25, *p *< .0001) and plant structures (*F *= 4.64, *df *= 2, 25, *p *= .019), but the interaction between these factors was not significant (*F *= 0.55, *df *= 4, 25, *p *= .69). After accounting for differences among plants structures, the concentration of Cry1A toxins was significantly higher in the Cry1Ac cultivar (33B) than in the Cry1Ac+Cry2Ab (Bollgard 2) and Cry1Ac/Cry1Ab cultivar (GK19) (Table 4). Overall, the concentration of Cry1A toxins was highest in leaves (mean* *= 0.27 μg/g fresh weight, SE* *= 0.02), intermediate in buds (0.23 μg/g, SE* *= 0.02), and smallest in bolls (0.20 μg/g fresh weight, SE* *= 0.02). Linear contrasts revealed significant differences between Cry1A concentration of leaves and bolls (*p *< .005), but Cry1A concentration did not differ significantly between leaves and buds or buds and bolls (*p *> .12). As in previous studies (Brévault et al., 2013; Sivasupramaniam et al., 2008), the concentration of Cry2Ab in plant structures of Cry1Ac+Cry2Ab cotton was about 2 order of magnitudes higher than the concentration of Cry1Ac (Table 4). The concentration of Cry2Ab did not differ significantly among plant structures (*F *= 2.38, *df *= 2, 9, *p *= .14).

**Table 4:** Concentrations of Cry1A and Cry2Ab toxins (SE in parentheses) in different Bt cultivars and plant structures

Cultivar	Plant structure	Cry1A (μg/g fresh weight)^a^	Cry2Ab (μg/g fresh weight)
Cry1Ac (33B)	Bud	0.29 (0.03)	
	Boll	0.27 (0.03)	
	Leaf	0.37 (0.03)	
	Mean concentration	0.31 (0.02)^1^	
Cry1Ac/Cry1Ab (GK19)	Bud	0.20 (0.03)	
	Boll	0.16 (0.03)	
	Leaf	0.20 (0.03)	
	Mean concentration	0.19 (0.02)^2^	
Cry1Ac+Cry2Ab (Bollgard 2)	Bud	0.21 (0.03)	43.81 (5.21)
	Boll	0.17 (0.03)	28.77 (5.21)
	Leaf	0.25 (0.03)	41.39 (5.21)
	Mean concentration	0.21 (0.02)^2^	37.99 (3.01)

a Mean concentration of Cry1A toxin followed by different numbers was significantly different (linear contrasts, *p *< .0008).

## 4 Discussion

Our results indicate that resistance to Cry2Ab in the An2Ab strain of *H. armigera* was nearly dominant, autosomally inherited, and controlled by more than one locus. As expected, results from diet overlay bioassays show that evolution of resistance to Cry2Ab resulted in strong cross‐resistance to Cry2Aa (81‐fold) and weaker but significant cross‐resistance to the Cry1A toxins (18‐ to 22‐fold). Such cross‐resistance between Cry2Ab and Cry1A increased survival of the An2Ab strain relative to the An strain on the Cry1A cultivars, showing that cross‐resistance conferred a selective advantage to Cry2Ab‐resistant individuals on Cry1A cotton. Results with Cry1Ac+Cry2Ab plants show that redundant killing was incomplete, as survival on this pyramid was significantly higher in An2Ab than in An. However, survival did not differ significantly between An and An2Ab on non‐Bt cotton, indicating an absence of fitness costs affecting this trait. These results indicate that none of the four conditions evaluated here, which are expected to influence success of the refuge strategy for Cry1Ac+Cry2Ab cotton, were met in the An2Ab strain of *H. armigera* from northern China.

The An2Ab strain analyzed here was founded using a F_2_ screen method, whereby a discriminating concentration of Cry2Ab was used to isolate 10 field‐derived lines carrying resistance alleles, which were then pooled and selected in the laboratory for resistance to Cry2Ab. Although such approach increases the likelihood of isolating field‐derived resistance alleles, further work will be needed to identify genes that confer resistance to Cry2Ab in An2Ab and confirm that mutations in these genes are present in field populations. The genetic basis of resistance to Cry1Ac has been well studied (Adang, Crickmore, & Jurat‐Fuentes, 2014; Tay et al., 2015; Wu, 2014), but relatively little is known about genetic changes conferring resistance to Cry2Ab in *H. armigera* or other insects (Tay et al., 2015). In *H. armigera* from Australia, a mutation in an ATP transporter gene (ABCA2) was tightly linked with resistance to Cry2Ab (Tay et al., 2015). The role of ABCA2 was revealed by analysis of the SP15 strain of *H. armigera*, which was produced by laboratory selection for resistance to Cry2Ab of a single line isolated with a F_2_ screen (Mahon et al., 2007). Interestingly, the mutation found in SP15 was also present in four *H. armigera* lines independently produced with F_2_ screens between 2002 and 2012, indicating that this mutation was present in field populations (Tay et al., 2015).

The dominance of resistance declines as a function of Bt toxin concentration (Tabashnik, Gould, & Carrière, 2004), implying that pests with low inherent susceptibility to Bt toxins are not expected to exhibit recessive resistance to Bt toxins (Carrière et al., 2010, 2015, 2016; Tabashnik et al., 2009, 2013). Previous studies of *H. armigera* strains from northern China reveal that some cadherin mutations and other mutations not linked to cadherin can confer nonrecessive resistance to Cry1Ac cotton (Jin et al., 2013, 2015; Zhang et al., 2012). In other studies of *H. armigera* from Australia, resistance to Cry1Ac cotton became less recessive as toxin concentration declined in older cotton plants (Bird & Akhurst, 2004, 2005). Resistance to Cry2Ab was recessive and conferred by a single locus in *H. armigera* from Australia (Mahon, Olsen, & Downes, 2008; Mahon et al., 2007; Tay et al., 2015). By contrast, results from this study indicate nearly dominant resistance to Cry2Ab in the An2Ab strain of *H. armigera*.

Although dominant resistance is expected to accelerate evolution of resistance to Bt crops relative to recessive resistance (Carrière & Tabashnik, 2001; Onstad & Meinke, 2010), the large natural refuges available in China are expected to delay resistance evolution even with nearly dominant resistance (Brévault, Nibouche, Achaleke, & Carrière, 2012; Heuberger, Crowder, Brévault, Tabashnik, & Carrière, 2011; Jin et al., 2015). Nevertheless, field monitoring of *H. armigera* resistance to Cry1Ac in northern China demonstrates that the frequency of alleles conferring nonrecessive resistance increased faster than the frequency of alleles conferring recessive resistance (Jin et al., 2015). As indicated here for Cry2Ab and shown elsewhere for Cry1Ac (Jin et al., 2013, 2015; Zhang et al., 2012), nonrecessive resistance to these toxins could be common in *H. armigera* populations from northern China. This raises concern about use of Cry1Ac+Cry2Ab cotton as a replacement for Cry1Ac cotton.

To assess the number of loci affecting resistance to Cry2Ab, method 1 relied on measured mortality of the F_1_ and An progeny to calculate expected responses of the backcross progeny (F_1_ × An). Results from this analysis provide strong evidence that more than one locus confer resistance to Cry2Ab in the An2Ab stain of *H. armigera*. By contrast, methods 2 and 3 relied on the assumptions of equal and additive effects of alleles and independently segregating loci to calculate expected responses of the backcross progeny or estimate the minimum number of independently segregating resistance genes, respectively (Lande, 1981; Tabashnik, 1991). Violation of these assumptions is expected to result in an overestimate of the number of loci affecting resistance (Tabashnik, 1991), implying that estimates obtained with methods 2 (i.e., five loci) and 3 (i.e., four or five loci) could be too high. Linkage map analyses are under way to refine these estimates and identify genes that confer resistance to Cry2Ab in An2Ab.

Because amino acid sequence similarity in domain II of Cry1A and Cry2Ab toxins is relatively low, cross‐resistance between these toxins is expected to be moderate (Carrière et al., 2015; Welch et al., 2015). Nevertheless, significant but low cross‐resistance between Cry1A and Cry2A toxins is generally present in pests targeted by Bt crops (Carrière et al., 2015), including *H. armigera* and the closely related noctuid *Helicoverpa zea* (Welch et al., 2015). As in another study performed with a different *H. armigera* strain collected in northern China (Wei et al., 2015), selection for resistance to Cry2Ab in the present study resulted in relatively high cross‐resistance to Cry1A toxins. By contrast, selection for resistance to Cry1Ac in strains of *H. armigera* originating from northern China resulted in lower cross‐resistance to Cry2A toxins (Jin et al., 2013; Luo et al., 2007; Xu et al., 2005; Yang et al., 2009; Zhang, Wu et al. 2012). Analysis of resistance ratios from these studies (after averaging RR for the three Cry1A toxins evaluated here) reveals that cross‐resistance between Cry1A and Cry2A toxins was significantly higher when selection was carried out with Cry2Ab (*n *= 2 cases, range* *= 20–61, back‐transformed RR* *= 34) than Cry1Ac (*n *= 8 cases, range* *= 1.0–6.8, back‐transformed RR* *= 2.1) (two‐sample *t*‐test on log‐transformed RR, *t *= 4.34, *p *= .0025). Similar to results obtained with *P. gossypiella* from Arizona (Tabashnik et al., 2009), this indicates asymmetrical cross‐resistance between Cry1A and Cry2A in *H. armigera* populations from northern China (Wei et al., 2015). Interestingly, such asymmetrical cross‐resistance does not appear to be present in *H. armigera* and *H. punctigera* from Australia, in which selection for resistance to Cry2Ab resulted in low cross‐resistance to Cry1A toxins (Caccia et al., 2010; Mahon et al., 2007).

Susceptible insects of pests with low inherent susceptibility to Bt toxins often show significant survival on pyramided Bt crops, implying that even low cross‐resistance in such pests should increase the selection differential between individuals with and without resistance alleles (Carrière et al., 2010, 2015, 2016). In experiments with single‐toxin cotton performed here, survival from neonate to pupation was significantly higher in An2Ab than in An on both Cry1A and Cry1Ac/Cry1Ab cotton. This provides the most direct support to date for the hypothesis that cross‐resistance between Cry toxins can contribute to evolution of resistance to Bt crops by increasing the selection differential between susceptible and resistant insects. The significant difference between the concentration of Cry1A toxins in the Cry1Ac (33B) and Cry1Ac/Cry1Ab cultivar (GK19) (Table 4) did not appear to have a marked influence on survival of the An and An2Ab strains (Table 3).

Redundant killing occurs when insects resistant to one Bt toxin are killed by another toxin in a pyramid (Brévault et al., 2013). Here, survival was significantly higher in An2Ab than in An on Cry1Ac+Cry2Ab cotton, showing that Cry1Ac produced in this pyramid did not kill all individuals resistant to Cry2Ab. A similar situation was observed in the GA‐R strain of *H. zea* selected for resistance to Cry1Ac: GA‐R survived significantly better than GA (the unselected strain) on Cry1Ac+Cry2Ab cotton (Brévault et al., 2013). Interestingly, survival of An2Ab was quite similar on Cry1Ac+Cry2Ab cotton and single‐toxin cotton (Table 3). This could indicate that efficacy of Cry2Ab against An2Ab was much reduced and increased survival of An2Ab on Cry1Ac+Cry2Ab cotton relative to An occurred primarily because cross‐resistance between Cry2Ab and Cry1Ac reduced efficacy of Cry1Ac.

The extent of redundant killing can be quantified using the redundant killing factor: RKF* *= 1 − [(proportion survival on pyramid for insects homozygous resistant to one toxin) − (proportion survival on pyramid for insects homozygous susceptible to both toxins)], with 0 indicating no redundant killing and 1 complete redundant killing (Brévault et al., 2013). In previous studies of *H. armigera* on Cry1Ac+Cry2Ab cotton, RKF was highest (0.98) in individuals feeding on presquare cotton and lowest (0.81) in individuals feeding on cotton with bolls (Carrière et al., 2015; Mahon & Olsen, 2009). RKF was 0.96 in the present study, indicating relatively low survival of An2Ab on pyramided cotton. RKF was 0.94 in *H. zea* feeding on cotton bearing bolls (Brévault et al., 2013). Survival and RKF estimates obtained here for An and An2Ab feeding on Cry1Ac+Cry2Ab cotton bearing bolls correspond more closely to survival and RKF estimates for the GR and SP15 strain of *H. armigera* feeding on early‐squaring Cry1Ac+Cry2Ab cotton (Mahon & Olsen, 2009). These differences could be related to between‐study variation in methods, strains, or cultivars.

Fitness costs associated with resistance to Cry1Ac cotton appear common in *H. armigera* (Bird & Akhurst, 2004, 2005; Cao et al., 2014; Liang et al., 2008). By contrast, no costs of resistance to Cry2Ab have so far been found in the SP15 strain from Australia (Mahon & Olsen, 2009; Mahon & Young, 2010). Here, survival to pupation on non‐Bt cotton was similar in An and An2Ab, indicating an absence of costs affecting this trait. In a review of 53 studies of insect resistance to Bt (Gassmann, Carrière, & Tabashnik, 2009), the percentage of comparisons in which significant survival costs were detected was 28%, which was lower than for some other traits (e.g., 49% development time; 46% female fecundity; 38% growth rate). Additional work will be needed to fully evaluate costs of resistance to Cry2Ab in the An2Ab strain.

Projections from simulation models that fit closely the temporal increases in frequency of Cry1Ac resistance in field populations of *H. armigera* indicate that >50% of Cry1Ac‐resistant individuals will be present in northern China by 2017 if conditions remain constant (Jin et al., 2015). Because of the necessary delay in producing enough seeds of pyramided Bt cotton to replace Cry1Ac cotton, it appears possible that Cry1Ac+Cry2Ab cotton could act as a single‐toxin crop (or less than that with cross‐resistance) when released in northern China. Furthermore, supporting findings from other studies (Gao, Wu, Gould, & Shen, 2009; Jin et al., 2013; Wei et al., 2015), our results indicate that several conditions underlying success of the refuge strategy for Cry1Ac+Cry2Ab cotton may not be met in *H. armigera* from northern China, which indicates that switching to this pyramid would not be the best option. Although Vip3Aa is unlikely to be effective against *P. gossypiella* (Tabashnik et al., 2012), three‐toxin pyramided cotton such as Cry1Ac+Cry2Ab+Vip3Aa, anticipated to be available in Australia in 2015–2016 and the United States in 2016–2017, could represent a better alternative for mitigating Bt resistance in *H. armigera*. Because weak but significant cross‐resistance is expected between Cry1A toxins and Vip3Aa even if these toxins have no similarity in amino acid similarity of domain II (Carrière et al., 2015; Welch et al., 2015), introduction of three‐toxin pyramids producing Vip3Aa could be unlikely to entirely eliminate the problem of cross‐resistance.

For pests with low inherent susceptibility to Bt toxins such as *H. armigera,* management tactics aimed at reducing the selection differential between individuals with and without resistance alleles (e.g., sprays of non‐Bt insecticides or cultural control applied in fields of Bt crops) should be considered for sustaining efficacy of pyramided Bt crops (Bates, Zhao, Roush, & Shelton, 2005; Carrière, Ellers‐Kirk, Pederson, Haller, & Antilla, 2001; Carrière, Sisterson, & Tabashnik, 2004; Carrière et al., 2016; Downes et al., 2010; Fitt, 2000; Fitt et al., 2004). Because single‐toxin crops act as stepping stones for resistance to pyramids, rapid and complete replacement of Cry1Ac cotton by pyramided cotton should be envisaged in China (Carrière et al., 2016). Such rapid switch between Cry1Ac and three‐toxin cotton could be challenging, as >30 companies dominate the cotton seed market and seed saving is common in cotton producers in China (Huang, Chen, Mi, Hu, & Osir, 2009).

## Data Archiving Statement

Data available from the Dryad Digital Repository: http://dx.doi.org/10.5061/dryad.fc8np.
