# Supplementary material for: Resistance to Bacillus thuringiensis toxin Cry2Ab and survival on single‐toxin and pyramided cotton in cotton bollworm from China
Source: Evol Appl. 2016 Dec 16;10(2):170–9. doi: 10.1111/eva.12438 (PMC5253426; doi:10.1111/eva.12438)
Supplement: Supplementary file 1 [file EVA-10-170-s001.docx]

**Supplementary Information**

**Table S1.** Observed vs. expected mortality at each of 10 concentrations of Cry2Ab in backcross generation (F_1_ × An) of *H. armigera*.

| Concn (μg/cm^2^) | Observed mortality | Expected mortality | | |
| --- | --- | --- | --- | --- |
|  |  | One locus | Two loci | Five loci |
| 0.03 | 0.042 | 0.101 | 0.064 | 0.047 |
| 0.06 | 0.063 | 0.155 | 0.105 | 0.083 |
| 0.125 | 0.125 | 0.224 | 0.165 | 0.140 |
| 0.25 | 0.188 | 0.296 | 0.258 | 0.215 |
| 0.5 | 0.271 | 0.370 | 0.325 | 0.307 |
| 1 | 0.354 | 0.444 | 0.422 | 0.414 |
| 2 | 0.583 | 0.517 | 0.524 | 0.527 |
| 4 | 0.625 | 0.591 | 0.625 | 0.637 |
| 8 | 0.750 | 0.665 | 0.718 | 0.738 |
| 16 | 0.896 | 0.739 | 0.798 | 0.822 |
| Mean difference (%) ^a^ |  | 53.1 | 24.2 | 12.1 |
| *X*^2 b^ |  | 22.73 | 7.84 | 4.06 |
| *P ^c^* |  | 0.0068 | 0.55 | 0.91 |

^a^ Mean difference calculated as the mean of the difference between observed and expected mortality (absolute values) across the concentrations tested.

^b^ Goodness of fit chi-square statistics evaluating deviation between observed and expected mortality across the concentrations tested (df = 9).

^c^ Probability indicating significant difference (P < 0.05) between observed and expected mortality.
